# Spore-Derived Isolates from a Single Basidiocarp of Bioluminescent *Omphalotus olivascens* Reveal Multifaceted Phenotypic and Physiological Variations

**DOI:** 10.3390/microorganisms13010059

**Published:** 2025-01-01

**Authors:** Rudy Diaz, David Bermudes

**Affiliations:** 1 Los Angeles Mycological Society, Los Angeles, CA 90025, USA; rudy@lamushrooms.org; 2 Department of Biology, College of Science and Math, California State University, Northridge, CA 91330, USA

**Keywords:** antibacterial agents, anticancer agents, bioluminescence, fluorescence, *Omphalotus olivascens* pigments, secondary metabolites

## Abstract

The fungal genus *Omphalotus* is noted for its bioluminescence and the production of biologically active secondary metabolites. We isolated 47 fungal strains of *Omphalotus olivascens* germinated from spores of a single mushroom. We first noted a high degree of variation in the outward appearances in radial growth and pigmentation among the cultures. Radial growth rates fell into at least five distinct categories, with only slower-growing isolates obtained compared with the parental dikaryon. Scanning UV-vis spectroscopy of liquid-grown cultures showed variation in pigmentation in both the absorption intensity and peak absorption wavelengths, indicating that some isolates vary from the parental strain in both pigment concentration and composition. Bioluminescence intensity was observed to have isolates with both greater and lesser intensities, while the increased emission in response to caffeic acid was inversely proportional to the unstimulated output. Under UV illumination, the media of the parental strain was observed to be brightly fluorescent, which was not due to the pigment, while the isolates also varied from greater to lesser intensity and in their peak emission. At least three separate fluorescent bands were observed by gel electrophoresis from one of the cultures, while only one was observed in others. In a subset of the cultures, fluorescence intensity varied significantly in response to casamino acids. None of this subset produced an antibiotic effective against *Staphylococcus aureus*, and only the haploids, but not the parental heterokaryon, produced an antibiotic consistent with illudin M effective against *Mycobacterium smegmatis*. This same subset produced an anticancer agent that was highly potent against MDA-MB-468 breast cancer tumor cells. We interpret these variations in haploids as significant in altering *Omphalotus* physiology and its production of secondary metabolites, which may in turn alter their ecology and life cycle, and could be further applied to studying fungal physiologies and facilitate linking them to their genetic underpinnings.

## 1. Introduction

The fungal genus *Omphalotus* (Fayod) has long been known as a source of bioactive secondary metabolites and is one of the limited numbers of bioluminescent fungi, thus this genus is of widespread interest. The species *Omphalotus olivascens*, H.E. Bigelow, O.K. Mill. and Thiers, is largely endemic to California and has not been extensively examined for its physiological and genetic potential.

Bioluminescence is a property shared by all the members of the genus Omphalotus [1], and its genes bear a unique genetic signature [2]. Fungal luminescence remained an enigma until the luciferin precursor, hispidin, was revealed by Purtov et al. [3,4]. Hispidin was subsequently shown to be recycled to caffeic acid [5]. Knowledge of the role of hispidin was instrumental in isolating its luciferase and in determining the complete biosynthetic pathway of the luciferin [6]. Notably, hispidin is fluorescent, with an excitation maxima of 380 and an emission of approximately 530, very similar to the emission maxima of fungal luminescence [7].

Earlier, other fluorescent compounds were thought to potentially be related to fungal bioluminescence. Ichihara et al. [8] isolated dihydroilludin S, and suggested a possible connection to bioluminescence. Endo et al. [9] determined the fluorescence of two compounds from *Omphalotus (Clitocybe) illudins*. It was noted that the fluorescence emission peak of an ergosta compound at 530 nm was very similar to the light emission peak of fungal bioluminescence. Additional fluorescent molecules were also found in *Omphalotus* mushrooms, and were considered as candidates for its luciferin. Isobe et al. isolated flavin [10], and then lampteroflavin [11], both with fluorescent emissions of 524 nm. This was noted to be similar to their measurements of mushroom bioluminescence emission. The possible relationship between flavin mononucleotide (FMN) and fungal bioluminescence seemed at the time to be supported by the earlier discovery of FMN, which is the luciferin in bacterial bioluminescence [12]. Later, the sesquiterpene panal was also proposed to be the mushroom luciferin [13]. Oxidized hispidin was only much later definitely determined to be the true luciferin as described above.

Other properties of members of this genus have also been noted. Early in the history of antibiotic discovery, mushrooms in Basidiomycota were among the sources investigated for their antibiotic potential [14,15,16,17,18,19,20,21,22,23,24]. Among these, Robbins et al. [14] began investigation of *Omphalotus illudens* (synonym *Clitocybe illudens*), work which continues today. Anchel et al. [21] found that extracts of these mushrooms showed activity against *Staphylococcus aureus* and *Mycobacterium smegmatis*. Chloroform extracts contained the anti-mycobacterial component, which concomitantly decreased in the aqueous phase, and was named illudin M. The substance remaining in the aqueous fraction was more strongly active against *S. aureus*, and was further extractable with ethyl acetate, and was named illudin S.

Nakanishi et al. demonstrated the antitumor activity of the compound lampterol, isolated from *Omphalotus guepiniiformis* (synonym *Lampteromyces japonicus*), against Ehrlich murine ascites tumors [25]. It was subsequently recognized that lampterol was identical with illudin S [21] when its chemical structure was determined [26,27,28,29]. Derivatives of illudins were later investigated for improved anticancer properties. It was shown that the acylfulvene illudin derivative hydroxymethylacylfulvene (irofulven) was selectively toxic against myelocytic leukemia and epidermoid carcinoma cells [30,31]. Irofulven was subsequently shown to display antitumor activity against the human xenograft lung carcinoma model MV522 [32,33]. More recently, conjugates of illudin M have been shown to have potent antitumor activity [34]. Improved production of illudin M has been investigated in order to supply material for larger studies [35,36,37].

Fungi in the phylum Basidiomycota typically have a life cycle in which sexual reproduction occurs with the formation of a basidiocarp (mushroom) that is haploid and dikaryotic, produced from a haploid dikaryotic mycelium. The fungus undergoes karyogamy only in its basidia in order to produce haploid basidiospores through meiosis [38]. These haploid spores are dispersed, often by the wind, and are capable of germinating to give rise to a haploid mycelium. In most Basidiomycetes, they must undergo fusion with a compatible haploid mycelium in order to be capable of completing their sexual life cycle. This sexual compatibility is mostly regulated by the heterozygosity of two mating type loci. These loci are linked in some systems and result in two mating types (bipolarity) and segregate independently in other systems, which results in four mating types (tetrapolarity) [39,40]. In the genus *Omphalotus*, tetrapolar sexuality has been described, with compatible monokaryons forming clamp connections [41]. Sub-variations in mating types can result in a greater range of incompatibilities, resulting in many more mating types [42,43]. Once established, a mycelium such as that of *Armillaria gallica* may remain in the haploid dikaryotic state for millennia [44], although there may also be many different genetic events that can occur among monokaryons and dikaryons to generate a variety of mosaic individuals [45,46,47,48,49]. There are also notable exceptions where a mycelium that produces a basidiocarp is not haploid dikaryotic, including homokaryotic fruiting in *Agrocybe aegerita* [50] and *Amanita phalloides* [51].

Much of what is known about Basidiomycetes originally became evident through the study of their sexually reproductive structures—the mushrooms they produce. Geographic distribution, species and/or biotype differences, associations with plants, toxicity, food and medicinal values, bioluminescence, and decomposition all contributed to our knowledge, derived from the study of the mushrooms alone. Knowledge of the mycelial basis of Basidiomycetes came first through their cultivation, eventually leading to a general understanding of their mode of reproduction.

Based on the prominence of Basidiomycete dikaryons and their fruiting bodies, there has been overall less study of their monokaryons. Of the areas of interest in monokaryons, mating type studies initially focused on *Schizophyllum commune* as an informative model [52], and haploids of other mushroom-forming species have been studied physiologically for edible mushroom production [53] and plant biomass degradation [54]. Peabody et al. [55] studied phenotypic differences in natural haploid isolates of *Armillaria gallica*. They found variation in rates of growth and in genetic plasticity in response to water potential. *A. gallica* was also revealed to generate a diploid state within the vegetative mycelium. Variation in bioluminescence intensity has been shown in spore-derived isolates of *Armillaria mellea* [56], and similar findings were earlier reported for *Panellus stipticus* (orth. var. *P. stypticus*) [57]. More recently, monokaryons of *S. commune* were studied and found to exhibit differences in lignocellulose degradation, culture morphology, fruiting body morphology, strain-specific carbon source profiles, and other physiological differences [58]. Genomic analysis of the haploid strains revealed unexpectedly large variances, with monokaryons exhibiting genetic variation of 11.2% across strains, with the genes for mushroom formation and lignocellulose being largely conserved.

Here, we describe some of the phenotypic variations found in basidiospore-derived isolates of *Omphalotus olivascens*. The survey included typical presumptive monokaryons that lacked clamp connections and a few heterokaryons that were obtained, as well as atypical highly branched isolates that lack clamp connections and grow very slowly. We show that basidiospore-derived isolates are a rich source of phenotypic variation in physiology and in the production of secondary metabolites, and suggest they could help link such traits to their genetic underpinnings.

## 2. Materials and Methods

### 2.1. Fungal Cultures

*O. olivascens* mushrooms were collected under the permission of the TreePeople Land Trust (TPLT). A spore print was collected from an individual fruiting body, and a tissue sample of the parental heterokaryon was obtained from the stipe trama. A voucher specimen was deposited in the University of California, Los Angeles, Herbarium (LA; fungarium col. #: DB.24.001). The spores were resuspended in sterile distilled water, vortexed vigorously, counted with a hemocytometer, and examined for clumping. The spores were subjected to a dilution series in sterile distilled H_2_O and then plated to malt extract agar (MEA; 2% malt extract with 2% agar and 30 μg/ mL chloramphenicol (_CAM30_) [41] and maintained under ambient laboratory conditions. Germinated isolates were collected between 5 and 17 days. Differences in the size of the cultures were noted and initially thought to represent possible early and late germination times, although we subsequently found there were genuine differences in the growth rates as described in our results. The isolates were transferred to bread crumb yeast agar (BCY with 1.5% agar on quadrant plates containing 30 μg/mL chloramphenicol; BCY_CAM30_). After adherence of the growing culture to the agar was observed (approx. 3–10 days), the plates were transferred to 4 °C for 2 months and subsequently photographed and subcultured. These and subsequent transferred culture materials were used for qualitative studies. For quantitative studies, the cultures were transferred to MEA and allowed to grow under ambient conditions, whereby the cultures were actively growing and had not reached the periphery of the Petri plate. Slower-growing cultures necessitated staggered starts to the culture inoculum. Uniform transfers were made all on the same day by utilizing the back end of a sterilized glass Pasteur pipette (approx. 4.5 mm diameter) to obtain a punch taken from the periphery of the actively growing cultures. Other fungal cultures used in this study were *Omphalotus olearius* Harold H Burdsall (HHB) 7441, *Omphalotus guepiniiformis* (*Lampteromyces japonicus*) Ronald H Petersen (RHP), 2305, *Panellus stipticus* (*P. stypticus*) American Type Culture Collection (ATCC; Manassas, VA, USA) 66462, *Armillaria mellea* (155798, Carolina Biological, Burlington, NC, USA), and *Pleurotus ostreatus* (Mueller’s Mushrooms, Alpine, CA, USA).

### 2.2. Growth Assessment

#### 2.2.1. Radial Growth

Radial growth was first surveyed visually among the isolates and given semiquantitative rankings of + (slowest growing), ++, +++, ++++, and +++++ (fastest growing). Representative isolates were quantitatively assessed by transferring four back-end Pasteur pipette plugs from the margin of the same actively growing cultures to separate 100 mm Petri dishes with MEA. Monokaryotic isolates (those lacking clamp connections described below) representing five a priori growth categories were compared with the parental isolate, as well as spore-derived heterokaryons. The cultures were grown at 25 °C in darkened conditions for 12 days, then photographed and measured. The diameter of an individual culture was calculated by taking the mean of the longest axis and the axis orthogonal. The difference in radial growth between groups of replicates was then analyzed with a one-way analysis of variance (ANOVA), followed by post hoc comparisons with Tukey’s HSD test.

#### 2.2.2. Mating Type Determinations

Mating trials were conducted for the first 12 isolates as described by Petersen and Hughes [41] using several different pairing approaches. Initially, four different isolates were separated by sterilized coverslip “windows” and a central plastic barrier (mahjong game tiles) to reduce the number of MEA plates needed. Faster-growing cultures with slow-growing cultures on the same plate sometimes resulted in overgrowth from neighboring cultures, and necessitated the further isolation of each pair of isolates per plate. The macro-morphology of the confrontations was scored as robust (compatible), barrage (same B mating type), flat (same A mating type), or overlapping (same A and B; no apparent reaction). The confronted hyphae were then observed with light microscopy (AmScope T340B compound microscope with MU500-HS digital camera and AmLite software v3.26.2023) directly through the coverslip on the media and scored for the presence or absence of clamp connections and the degree of branching.

### 2.3. Pigmentation

Variations in the pigmentation of cultures grown on BCY agar were visually notable. Pigmentation was further assessed in clear M9 minimal media at pH 6.8 (Difco, Becton Dickinson, Franklin Lakes, NJ, USA) with 0.4% glucose. Cultures of varying growth rates were allowed to grow for 2–4 weeks until pigmentation was visibly detectable. UV-Vis spectroscopy was performed on the culture supernatants that were spun at 17,000× *g* for 2 min. Separate scans were performed from 225 to 350 and 350 to 600 nm with a SpectraMax M3 (Molecular Devices, San Jose, CA, USA).

### 2.4. Bioluminescence

Luminescence was measured by luminometry as relative light units (REUs) with an Invitrogen iBright 1500 and using iBright Analysis software version 5.3.0 (Invitrogen/ThermoFisher Scientific, Waltham, MA, USA). The isolates were cultured in BCY_CAM30_ within black 24-well plates, with the cultures transferred as 1–2 mm pieces obtained by cutting actively growing mycelia with a scalpel. The non-luminescent *Pleurotus ostreatus* was used as a control. The cultures were then grown for 7 days with a diurnal cycle in ambient conditions (approx. 25 °C). Phenotypic variation in the response to caffeic acid was determined by its addition to cultures on the 7th day, applied with 100 μL of 1 mM caffeic acid (Pure Health Solutions, eBay) and 0.1% DMSO diluted from a 10-fold concentrated stock that was filter-sterilized (Puradisc 25 mm PES Syringe Filter, 0.2 µm).

### 2.5. Fluorescence

#### 2.5.1. Fluorescence in Minimal Media

The fluorescence of the culture media of the parental heterokaryon and other isolates growing on BCY media was noted using a hand-held 365 nm UV lamp. A qualitative comparison of the strains was assessed in 24-well black tissue culture plates with clear bottoms containing translucent M9-glucose agar. The plates were subjected to UV transillumination at 365 nm and photographed. The fluorescence excitation and emission of culture supernatants grown in clear M9 liquid media with glucose were determined from culture supernatants comparing the parental heterokaryon with notably fluorescent isolates that gave slightly different colored fluorescence. Scanning fluorimetry was used to determine the excitation and emission maxima with a SpectraMax M3. The M9 supernatants were also concentrated 10-fold by subjecting them to evaporation at low pressure, and then separated in a 2% agarose gel with 1X M9 salts as the buffer with 200 mA of current and photographed using 365 nm transillumination.

The fluorescent supernatants were subjected to organic solvent extraction by the addition of an equal volumes of either chloroform or ethyl acetate, followed by vortexing and centrifugation. The supernatants were also heated to 90 °C in a heat block.

#### 2.5.2. Growth and Fluorescence with Casamino Acids

The parental strain and non-clamped isolates pre-screened on 0.1% peptone agar were assayed for relative growth and fluorescence on four preparations of minimal media with 1.5% agar varying in composition of I, 0.2% glucose; II, 0.2% glucose, 0.1% KH_2_PO_4_, and 0.1% MgSO_4_ (from an autoclaved 10% stock); III, 0.2% glucose, 0.1% KH_2_PO_4_, 0.1% MgSO_4_, and 0.1% casamino acids; and IV, 0.2% glucose, and 0.1% casamino acids only. X-plates with quadrants containing each of the four media were inoculated with plugs from the margins of the same inoculum grown on MEA. These cultures were then grown at 25 °C in darkened conditions for 10 days. They were then viewed with 365 nm transillumination and photographed. The relative growth between treatments was scored as more robust (+), less robust (-), or no contrast (o). Relative fluorescence unit (RFU) intensity was analyzed quantitatively with ImageJ (NIH) by first splitting the UV images into RGB channels, then with the green channel, selecting three regions (50-pixel diameter) from uncolonized corners within each quadrant of the X-plate. Intensity in each region was measured as the mean gray value (sum of values divided by number of pixels). Correcting for the autofluorescence of blank media, differences in fluorescence intensity were analyzed with one-way ANOVA and Tukey contrasts.

### 2.6. Antibiological Activity

#### 2.6.1. Antibacterial Activity

Antibacterial disk diffusion assays were performed using fungal M9-glucose culture supernatants. In total, 10 μL of filter-sterilized (0.2 μm Whatman (ThermoFisher, Waltham, MA, USA) Puradisc 0.2 μm PES membrane) culture supernatants were absorbed into sterile 6 mm blank filter paper disks. Confluent bacterial cultures of *Staphylococcus aureus* (ATCC 29523) and *Mycobacterium smegmatis* (ATCC 14468) were inoculated onto tryptic soy agar (Difco), the culture supernatant disks applied on top, and then incubated at 37 °C overnight. The plates were photographed and the diameter of the zones of clearing was determined.

#### 2.6.2. Antitumor Cell Activity

The MDA-MB-468 human breast adenocarcinoma was obtained from the ATCC (MDA-MB- 468 was kindly provided by Dr. Jonathan Kelber, Baylor University), and was authenticated at the University of Arizona Genetics Core and determined to be mycoplasma-free (IDEXX Laboratories, Westbrook, ME, USA). Fungal culture supernatants were used to assess cytotoxicity toward MDA-MB-468. MDA-MB-468 cells were cultured in Dulbecco’s Modified Eagle Medium (DMEM), supplemented with heat-inactivated fetal bovine serum (10% vol/vol; Gibco/Life Technologies, Grand Island, NY, USA) and 1% penicillin–streptomycin. The cells were trypsinized for use in passages and seeding cell proliferation assays. Cancer cell survival was determined by RFUs using Alamar Blue (R&D Systems, Minneapolis, MN, USA) at 544/590 nm, using a SpectraMax M3 (Molecular Devices, Sunnydale, CA, USA) running SoftmaxPro 7.0.

## 3. Results and Discussion

All of the physiological/phenotypic characteristics we assessed varied substantially, and are summarized in Table 1 and described in detail in the sections below.

### 3.1. Fungal Cultures

**Figure 1:**
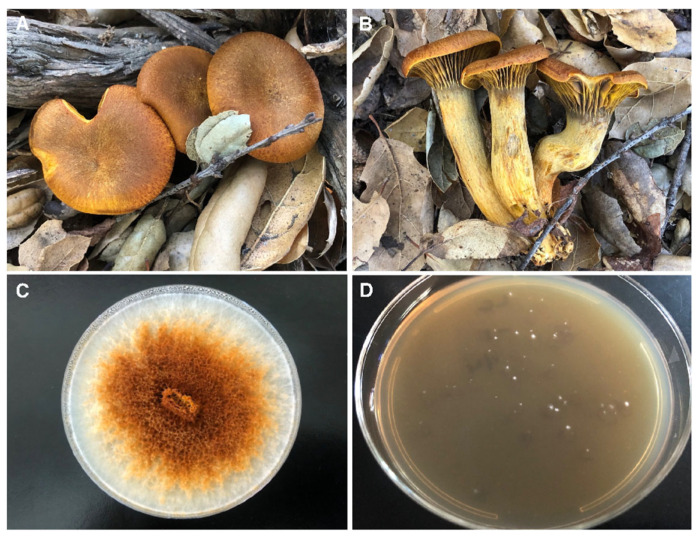
*Omphalotus olivascens* basidiocarps, the heterokaryotic culture obtained from mushroom tissue, and germinants. (**A**,**B**) *O. olivascens* was collected and a spore print obtained. (**C**) The mushroom was dissected aseptically and trama from the stipe was grown on BCY_CAM30_ (strain CC1). (**D**) The spores were then subjected to limiting dilution and germinated on MEA_CAM30_.

The mushroom that was collected and its tissue, sampled successfully, grew on MEA _CAM30_, and the spores germinated (Figure 1).

**Figure 2:**
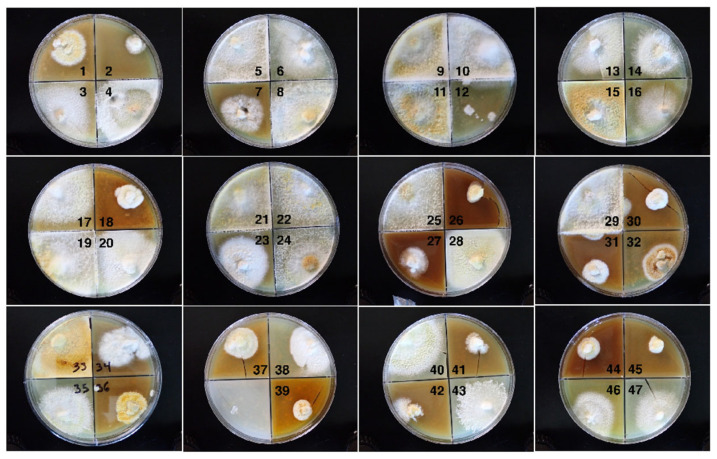
Spore-derived isolates 1–47. The isolates were transferred to quadrant plates containing BCY_CAM30_.

Along with the parental tissue isolate that had a rusty brown color somewhat similar to the mushrooms themselves, 47 basidiospore-derived isolates were included in this study (Figure 2; Table 1). Clone CC1-7, which initially grew slowly, exhibited a faster-growing sector, and the slower-growing component most similar to the initial strain was reisolated and designated CC1-7a. Clamp connections were observed in the hyphae of the parental isolate as well as seven spore-derived isolates (CC1-6, 14, 15, 20, 28, 33, 39), suggesting a heterokaryotic status. All other isolates did not possess clamp connections.

### 3.2. Growth

#### 3.2.1. Variation in Radial Growth

**Figure 3:**
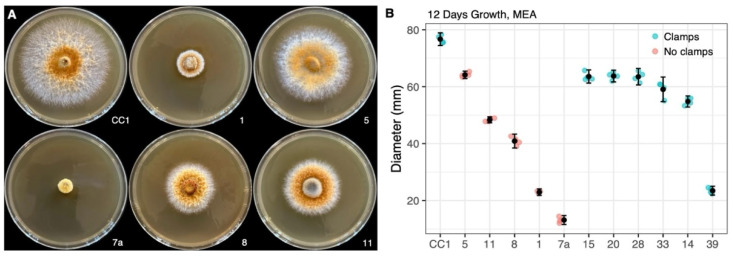
Variation in mycelial growth on MEA_CAM30_. (**A**) Growth across the parental strain (CC1) and five representative non-clamped isolates derived from basidiospores (CC1-1, 5, 7a, 8 and 11). (**B**) Difference in mean growth across the clamped (blue) and non-clamped isolates (red). Additionally, 95% confidence intervals are shown based on four replicates measured for all isolates except CC1-1, which had three.

Radial growth on MEA was highly consistent for replicates of the parental strain and non-clamped, range-representative isolates (Figure 3A), which showed a significant difference after 12 days. Based on statistical analysis (one-way ANOVA, F(5, 17) = 1932.34, *p* < 0.001), we found that the cultures varied significantly in mean diameter from 76 mm in the parental strain to 13 mm in CC1-7a (Figure 3B; four replicates were measured for all isolates except CC1-1, which lost one due to contamination). Compared to the parental heterokaryotic strain, all spore-derived clamped isolates measured (N = 6) showed significantly less radial growth (Tukey’s test, *p* < 0.001), ranging from 54 to 63 mm in five of these isolates (CC1-15, 20, 28, 33, and 14), to 23 mm in CC1-39 (Figure 3B). CC1-6, another clamped isolate, was omitted from radial growth assessment due to reproducibly switching its growth from slow-growing white mycelia to fast-growing rusty brown mycelia of various intensities. The pigmentation of mycelia varied with other factors based on age and room temperature vs. cold temperatures, but was not comparatively assessed.

The observation that all spore-derived isolates, including heterokaryons, achieve only as much as 82% of the radial growth of the parental isolate could be consistent with physiology under selection for outcrossing; although, differences in the nutrient composition of media can yield different inferences about natural physiology in Agaricomycetes [59].

The significance of these findings could be multifold. First, the relationship of such significant physiological variation to the ecology and life cycle of Basidiomycetes must be considered as the haploid stage constitutes its initial stage in the environment. The roles of each of these physiological features outside the laboratory environment could play a role in the survival of haploids in different environmental conditions and at different times, as well as in sexual competition across haploids for limited mating partners [60].

#### 3.2.2. Mating Types

**Figure 4:**
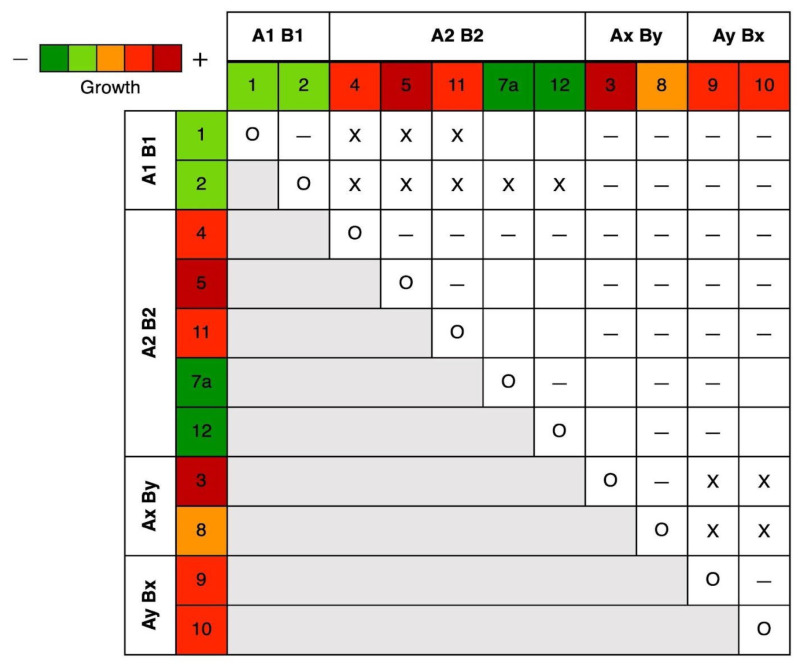
Mating trials for the first 12 isolates, with colors indicating pre-observed growth rates. Pairings were scored as self (O), mating compatible (X), not compatible (–), or not observed (blank).

**Figure 5:**
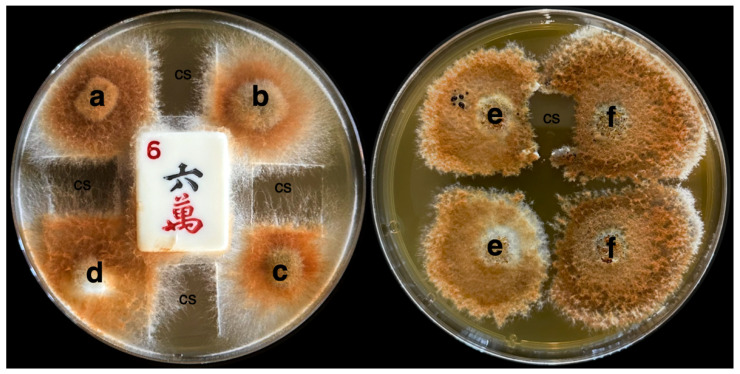
Plating setup for mating type pairing experiments. (**Left**) Up to four different strains (a–d) can be paired on one plate using a barrier (sterile mahjong game tile). Observation of the resulting hyphae produced by the pairing is further facilitated by the placement of a sterile coverslip (cs) “window”. Faster-growing strains, however, have the potential to scale the barrier. (**Right**) Pairings of the same two strains (e,f), with and without a window. These plates facilitate both observation of the hyphae and the overall mating type reaction, especially for slower-growing strains.

A heterothallic tetrapolar system was observed on MEA, consistent with past studies of *Omphalotus* [41]. All four mating types were identified from non-clamped individuals among the first 12 isolates (Figure 4), with each mating type represented by at least two isolates. Examples of pairings with four strains per plate using barriers (mahjong tiles) with coverslip windows, and with only two strains per plate with only a single coverslip, are shown in Figure 5. Between isolates, heterozygosity at both mating loci was determined from compatible matings, which resulted in the formation of clamp connections in confronted hyphae for each of the studied individuals. Two isolates were determined homozygous at both loci if they showed compatible matings when paired with the same partner. After arbitrarily designating the first two mating types A1B1 and A2B2, isolates which did not show compatible matings with either of these first two were inferred to have mating types A1B2 or A2B1; however, morphological variability between confrontations of these non-mating isolates left us unable to resolve their status as either A or B locus incompatibilities. As such, the second set of complementary mating types is reported here as including AxBy and AyBx. Compatible matings in the slowest-growing isolates (CC1-7a and CC1-12) were observed with pairings against an isolate from each of the four determined mating types.

### 3.3. Variation in Pigmentation

**Figure 6:**
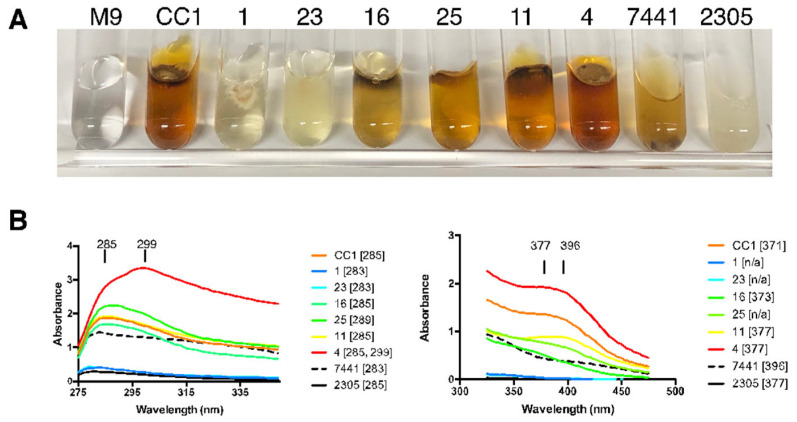
UV-Vis spectrum of isolates producing pigment in M9-glucose_CAM30_ liquid minimal media. (**A**) The M9-glucose control and the parental CC1 are shown next to a gradation of pigment intensity (CC1-1, 23, 16, 25, 11, 4) and *Omphalotus olearius* (HHB 7441) and *Omphalotus guepiniiformis* (RHP 2305). (**B**) Representative scans of the pigmentation visible when growing in liquid. Absorption peaks are given in brackets.

Pigmentation varied in liquid media and did not follow the same pattern on BCY agar as shown in M9-glucose (Figure 6; Table 1). This is exemplified by isolate #4, which was nearly white on BCY agar, but was the darkest grown in M9-glucose broth. We also noted other variations in pigmentation based on age and room temperature vs. cold temperatures, but did not comparatively assess them. The ability to separate the culture from the initially clear supernatant made the use of this media preferable, and it seems likely that it would vary in yet another medium and/or other carbon sources. The M9-glucose control and the parental CC1 are shown next to a gradation of pigment intensity (CC1-1, 23, 16, 25, 11, 4) as well as *Omphalotus olearius* (HHB 7441) and *Omphalotus guepiniiformis* (RHP 2305) (Figure 6A). In the shorter wavelength scan (Figure 6B, left side), there was a predominant peak at 285, with only CC1-4 showing a stronger additional peak at 299, indicating at least one additional pigment in high concentration. In the longer wavelength scan (Figure 6B, right side), a peak at 377 was the most prominent amongst the isolates, but with the parent having a peak at 371. *Omphalotus olearius* (HHB 7441) was the most different, with a peak at 396.

Based on these observations, pigmentation intensity is highly variable among isolates, and is differentially expressed when grown in different culture media. Although purified pigments would be required to precisely describe these differences, it is clear that CC1-4 makes an additional prominent pigment with an absorption peak at 299 and that minor variation occurred among the other isolates. These data also show that *O. olivascens* pigmentation is similar to *O. guepiniiformis*, and that *O. olearius* is not similar to these other two species when assessed in M9-glucose. Numerous pigments in fungi and bacteria have been studied (see Gill [61] and Ramesh et al. [62] for reviews), and several pigments in *Omphalotus* have been identified [63]. Pigments from *O. olivascens* are commonly used for dyeing fabrics, and differences in pigment production across haploids from another dye mushroom, *Phaeolus schweinitzii,* have also recently been noted (Sidnee Ober-Singleton, personal communication 2024).

### 3.4. Bioluminescence

#### 3.4.1. Variation in Luminosity

**Figure 7:**
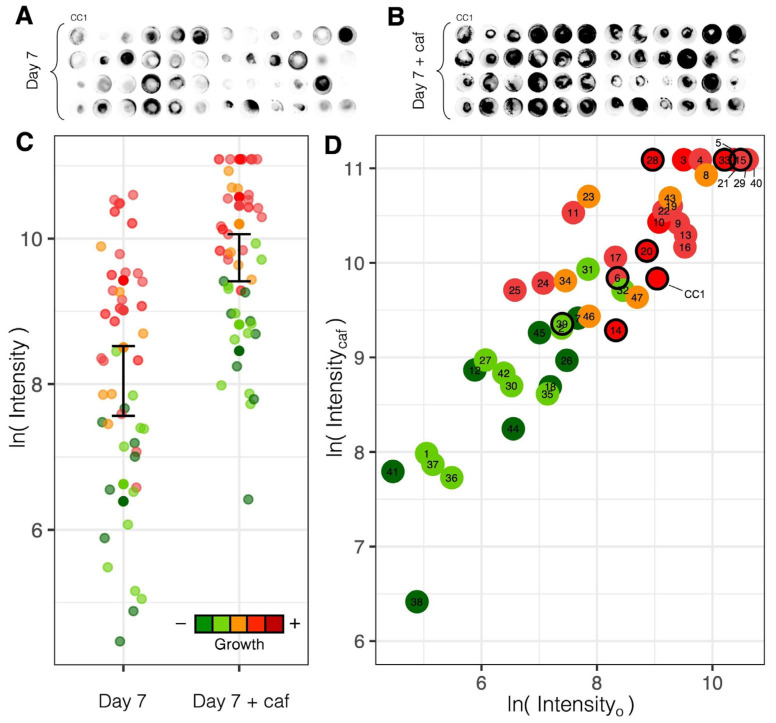
Bioluminescence across *O. olivascens* isolates. (**A**) The parental strain (CC1) and all 47 basidiospore-derived isolates, imaged collectively after 7 days of growth on BCY in two 24-well plates. (**B**) The same cultures imaged immediately after the addition of caffeic acid. (**C**) Difference in luminescence intensity (natural log-transformed) before and after treatment with caffeic acid, with 95% confidence intervals for the collective mean. (**D**) Enhancement in luminescence intensity (natural log-transformed) in each isolate after treatment with caffeic acid; black circles indicate isolates with clamp connections. Colors in C and D reflect pre-observed growth rate categories.

All *O. olivascens* isolates (N = 48) exhibited luminosity on BCY greater than background noise measured in the non-luminescent control species *Pleurotus ostreatus*. These luminous isolates varied from 460- to over 800-fold between the highest and lowest intensity units within three replicate sets started on different days, with several isolates showing consistently higher luminosity (e.g., at least 9000 more units) or lower luminosity (e.g., at least 5000 fewer units) than the parental isolate (median 8461 units; Table 1; Figure 7A). The top five isolates ranked across replicates by mean luminescence intensity (CC1-21, 4, 22, 5, and 10) were among the top one-third most luminous isolates in each replicate, and were brighter than the parent culture by as much as eight-fold. All five of these isolates lacked clamp connections and were scored a priori as belonging to the two fastest growth classes. The five isolates ranked last by mean luminescence (CC1-41, 36, 12, 35, and 1) were among the least luminous two-thirds of the isolates in each replicate, and showed an average luminosity dimmer than the parental isolate by 30- to 600-fold. All of the least luminous isolates also lacked clamp connections, and belonged to the two slowest-growth classes.

It was generally observed that the mycelia of fast-growing isolates had more volume and were more rusty brown than the mycelia of slow-growing isolates, which were whiter. Overall, the top 50% of cultures ranked by luminosity mostly contained isolates scored a priori as faster-growing (Figure 7C), though it was not strictly observed that luminosity corresponded to growth rate; several spore-derived isolates reproducibly showed greater luminescence than the parental strain, which was the isolate that exhibited the fastest growth on MEA.

These observations are consistent with both known classical genetics and the emerging feature(s) of fungal genomics, whereby the genetic content of haploids varies significantly [58]. The processes of crossing over, random assortment, and dominance and/or recessive traits being pronounced in haploids is consistent with our findings. Indeed, genetic loci associated with agronomic traits have been identified through segregation analysis in other mushroom-forming fungi [64,65,66,67]. However, with the backdrop of larger-than-expected heterozygosity becoming apparent in fungal genomes, we suspect that genomic analysis of *Omphalotus* haploids may reveal significant variations in DNA content in future studies. By comparison among haploids, increases in the bioluminescence or other characteristics could reveal the existence of suppressive regulatory elements, inhibitors or competing biosynthetic pathways, while decreases in bioluminescence could represent increased regulatory suppression, lack of activators, promoter elements, or cofactors not yet known. Thus, this approach provides an opportunity to link physiological functions to their corresponding genetic components.

#### 3.4.2. Response to Caffeic Acid

The functional response of the fungal bioluminescence pathway [6] was assessed across isolates through stimulation with caffeic acid, which is a biosynthetic component for the luciferin precursor hispidin [5]. The addition of caffeic acid enhanced the luminescence intensity of mycelia in all *O. olivascens* isolates (Figure 7B,C; Table 1), collectively showing a significantly greater mean luminescence intensity immediately after the treatment (Welch’s *t* test: t = −5.8807, df = 82.383, *p* < 1 × 10^−7^). A negative control of 100 μL of 0.1% DMSO without caffeic acid yielded an insignificant difference in measured luminescence after application in six replicate cultures of the parental strain (Welch’s *t* test: t = 1.1253, df = 9.8644, *p* > 0.2). The magnitude of the luminescence response to caffeic acid ranged from a 1.6- to 27-fold increase in luminosity, with dimmer isolates showing a greater increase in luminosity than brighter isolates. A regression analysis revealed a strong linear relationship in log-transformed luminescence intensity before and after treatment (Figure 7D; adjusted R^2^ = 0.823, *p* < 1 × 10^−18^), indicating that initial luminescence intensity is a strong predictor of intensity post-treatment.

Altogether, the parental strain and derived isolates studied here show luminosity that is predictably stimulated through the application of caffeic acid, consistent with known functions underlying fungal bioluminescence [5], and with isolates of higher baseline luminosity, showing diminishing returns suggestive of saturation. The consistent functional response observed across *O. olivascens* isolates is in line with the view that fungal bioluminescence may serve in one or more adaptive roles that are not well defined, which likely varies across species [1]. In *Omphalotus*, environmental stresses were found to induce differential transcriptional regulation of bioluminescence in *O. guepiniiformis* [68], though biological interpretation of such responses remains unclear.

### 3.5. Variation in Fluorescence

#### 3.5.1. Fluorescence Variation in Minimal Media

**Figure 8:**
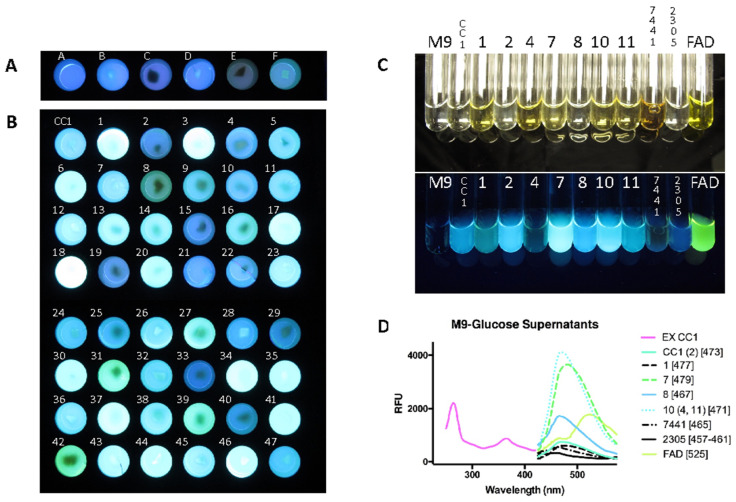
Fluorescence of fungal cultures. Cultures were transferred to 24-well plates with clear glass bottoms containing translucent M9-glucose agar and grown for 7 days. (**Panel A**) A, empty well; B, *Pleurotus ostreatus*; C, *Armillaria mellea*; D, *Panellus stipticus*; E, *Omphalotus olearius*; and F, *Omphalotus guepiniiformis* photographed with UV 365 nm transillumination; (**Panel B**) *O. olivascens* isolates CC1 (parental heterokaryon) and the spore-derived isolates 1–47 photographed with UV 365 nm transillumination. (**Panel C**) Representative culture supernatants (CC1, 1, 2, 4, 7a, 8, 10, 11) and *Omphalotus olearius* (7441), *Omphalotus guepiniiformis* (2305), and flavin adenine dinucleotide (FAD) in glass tubes photographed in light (top) and with UV 365 nm transillumination; the M9-glucose media was used as a control. (**Panel D**) The excitation spectrum of the CC1 supernatant and the emission spectra of the same representative cultures. A representative culture number given first, with the other cultures having the same peaks in parentheses, and the emission peaks given in brackets.

Variation in fluorescence was notable among Petri plates using a hand-held UV light, and growth in 24-well plates with M9-glucose agar allowed all the cultures to be observed simultaneously (Figure 8A,B). The empty well (Figure 8A, well A) exhibited the color of the 365 transillumination passing through the translucent M9-glucose agar. Among the comparator fungal species in Figure 8A, only *Armillaria mellea* (well C) showed any observable variation from the media-only well, which may have also had a contribution from non-fluorescent pigmentation. Variation between the parental strain (CC1) and isolates occurred in both fluorescence intensity and in minor color variations (Figure 8B). These fluorescent color variations were further apparent in M9-glucose culture supernatants of representative strains (Figure 8C). The fluorescence excitation and emission peaks were determined for these strains and showed variations consistent with their apparent minor color shifts, e.g., with culture CC1-8 appearing bluish (Em: 467 nm) and culture CC1-7a appearing more greenish (Em: 479 nm). The other two *Omphalotus* species (7441 and 2305) were bluer isolates than the parental strain or any of the isolates.

We further separated the fluorescent supernatants by agarose gel electrophoresis (Figure S1). A major bright band that was similar in migration rate was observed in all but CC1-4 and *O. olearius* and *O. guepiniiformis* (Figure S2A). Clone CC1-7a was the brightest following electrophoresis, and was further separated and showed at least three distinct bands (Figure S1B) that were not as readily apparent in the other strains. Because their observation may be due to concentration rather than their complete presence or absence, we only conclude that variation in the observable band composition occurred, and may account for the differences in peak fluorescence emissions.

Fluorescent compounds have previously been isolated from *Omphalotus* spp., including illudin S an ergosta [9], flavin [10], lampteroflavin [11], and panal [13]. The fluorescence substances in the *O. olivascens* culture supernatant were observed not to be diminished by 10 min at 90 °C (Figure S2), which was confirmed by fluorimetry. The substances in our study were not extracted with chloroform or ethyl acetate (Figure S2). These results show the lack of overall heat sensitivity of the fluorescent components, their lack of being readily volatile, and their insolubility in organic solvents. These features are not consistent with the known fluorescent compounds of bioluminescent fungi described by Endo et al. [9] or Nakamura et al. [13]. The absence of the characteristic flavin peak at 525 also indicates the *O. olivascens* fluorescent compounds are not the flavins FMN or lampteroflavin previously found in *Omphalotus guepiniiformis* [10,11]. Other known fluorescent compounds from mushrooms such as aurantricholides A and B isolated from *Tricholoma aurantium* are also extractable in organic solvents, and are unstable [69], so are not consistent with the fluorescent compounds in *O. olivascens* culture supernatants. The compounds we observed may represent new fluorescent substances, or known substances for which fluorescence has not been described. Their secretion into the culture supernatant which separates them from the mycelium, their water solubility, and their ability to be separated by electrophoresis should facilitate their purification and the elucidation of their chemical structures.

#### 3.5.2. Fluorescence and Growth Variation in Response to Casamino Acids

**Figure 9:**
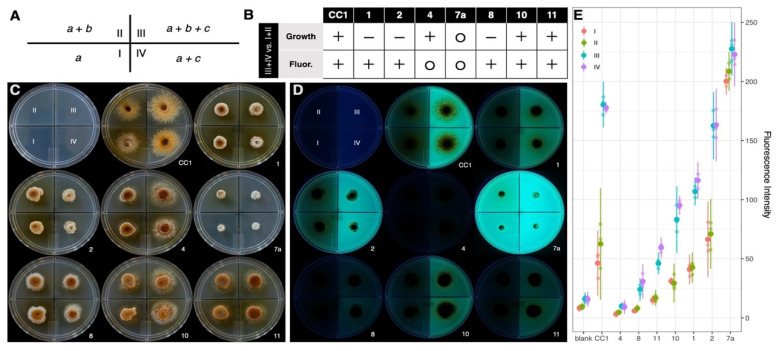
Phenotypes in minimal media with varied nitrogen. (**A**) Schematic of experimental design, with media composition based on (*a*) glucose, (*b*) inorganic salts, and (*c*) casamino acids. (**B**) Qualitative phenotypic contrasts between treatments with and without casamino acids; isolates are scored more (+), less (-), or no contrast (o) in mycelial growth and RFU of media with UV light (365 nm). (**C**) Cultures after 10 days of growth. (**D**) Fluorescence with UV transillumination. (**E**) Fluorescence intensity between treatments and across isolates, with 95% confidence intervals for three sampled regions in each treatment.

Following the preliminary observation of differential stimulation with peptone, phenotypes for the parental strain and non-clamped isolates with representative variation (N = 8) were scored in four treatments of minimal media with and without casamino acids and inorganic salts (Figure 9A). After 10 days of growth, discrete contrasts were apparent between treatments with and without casamino acids for phenotypes, including mycelial growth and media fluorescence (Figure 9B), which were consistent in a biological replicate created on a separate day. The parental heterokaryon and three derived isolates (CC1-4, 10, 11) showed more robust growth with casamino acids, while three other isolates (CC1-1, 2, 8) showed less robust growth (Figure 9C); none of these showed contrasts in growth with inorganic salts. The isolate belonging to the slowest a priori growth category (CC1-7a) showed the least growth in each treatment for all isolates, and showed no apparent growth contrast between treatments after 10 days.

The RFUs for media of a given treatment varied widely across strains (Figure 9D,E), and differed as much as 60-fold between the most and least intense. The RFUs also differed significantly between treatments within all strains (one-way ANOVA, *p* < 0.001), except for CC1-4 and CC1-7a (*p* > 0.05). For the strains with significant fluorescence contrast, treatments with casamino acids (III and IV) showed greater intensity than treatments without casamino acids (I and II; Tukey’s test, *p* < 0.05). Notably, CC1-7a had greater RFU intensity in its treatments without casamino acids than all other isolates showed in treatments with casamino acids. CC1-4 was the only isolate to measure lower fluorescence intensity than the blank media.

On the whole, growth and fluorescence are differentially inducible through the addition of a complex organic nitrogen source, suggesting differences in nitrogen metabolism. This result is consistent with the finding in another mushroom-forming species, the ectomycorrhizal *Tricholoma matsutake*, that nitrogen metabolism varies considerably across basidiospore-derived isolates generated from a single basidiocarp, suggesting variation in ecophysiology [53]. In an ecological context, the response to a complex organic nitrogen source could lead to selective pressure between individuals if their substrate is homogeneous in nitrogen composition, or foster complementary interactions between individuals if the substrate is heterogeneous [70].

### 3.6. Antibiological Activity

#### 3.6.1. Antibacterial Activity

**Figure 10:**
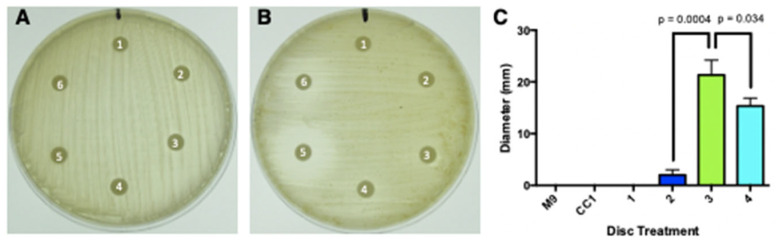
Disk diffusion assay of *O. olivascens* M9-glucose culture supernatants. (**A**) *Staphylococcus aureus* exposed to 1 (M9); 2 (CC1 heterokaryon); 3 (CC1-1); 4 (CC1-2), 5 (CC1-3), and 6 (CC1-4). (**B**) *Mycobacterium smegmatis* exposed to 1 (M9), 2 (CC1 heterokaryon), 3 (CC1-1), 4 (CC1-2), 5 (CC1-3), and 6 (CC1-4). (**C**) Graphical representation of the diameters measured for *M. smegmatis*.

Of the five culture supernatants compared with the M9-glucose media control, none were antibiotically active against *S. aureus* (Figure 10A). However, isolates CC1-3 and CC1-4 consistently had observable zones of inhibition against *M. smegmatis*, with only very minor inhibition from CC1-2, and none from the parental CC1 or CC1-1 (Figure 10B). The inhibition zones of those that were measurable were also statistically different from each other (Figure 10C). Based upon the bacterial sensitivity pattern established by Anchel et al. [21], these results indicate that *O. olivascens* makes detectable illudin M, but not illudin S, which was apparent only from the monokaryons and not the parental heterokaryon.

#### 3.6.2. Antitumor Cell Activity

**Figure 11:**
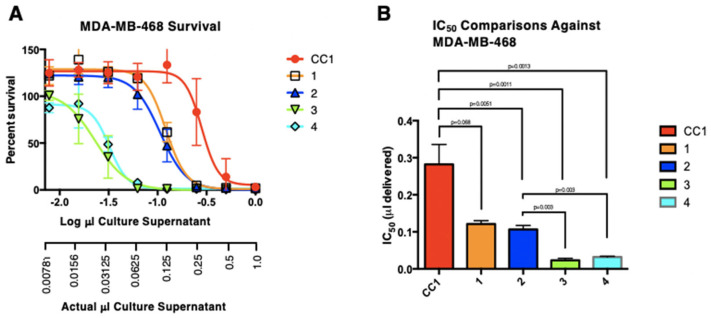
(**A**) Dose-response curves of *Omphalotus* isolate culture supernatants with error expressed as the standard error of the mean (SEM). Dose responses were determined as relative percent survival of MDA-MB-468 triple negative breast cancer cells following exposure to two-fold dilutions initiated from a 1:10 dilution of the respective *Omphalotus* cultures. (**B**) Comparison of the IC_50_ values and their significance.

Variation occurred in the μL IC_50_ values of the parental heterokaryon (CC1) and the first four monokaryons (Figure 11). CC1 had the highest (least potent) IC_50_ (0.28 μL ± 0.031), followed by CC1-1 and CC1-2 with similar IC_50_ values (0.12 μL ± 0.0051 and 0.11 μL ± 0.0063) and then CC1-4 and CC1-3 with IC_50_ values of 0.032 μL (±0.0018) and 0.023 μL (±0.0032), respectively. The CC1 heterokaryon was significantly less toxic than all the other isolates, while isolates CC1-1 and CC1-2 grouped together and were not significantly different from each other, but were significantly different from isolates CC1-3 and CC1-4, which also did not differ significantly from each other and were the most potent.

As with other behaviors of the isolates, cytotoxicity showed strong physiological variation. While only four of the isolates were compared with the parental heterokaryon, the most potent of these was approximately 10-fold greater than the parental strain. Based upon the antibacterial activity, the active component is consistent with illudin M. These antibiological activity studies were the only ones where only greater activity found in antibacterial and anticancer agent production. In all other cases, pigmentation, bioluminescence, response to caffeic acid, fluorescence, and responses to casamino acids, significantly greater and lesser quantitative properties were obtained. Such variations, alone and in combination, can be considered emergent properties relative to the parental strain.

Although promising, the anticancer candidate irofulven did not progress past phase II clinical studies in humans [71]. However, Kim et al. [2] noted that there is renewed interest in illudins because of the enhanced sensitivity in cancers with mutations in the transcription-coupled nucleotide-excision repair (TC-NER) pathway. They also noted that genetic modifications to increase production of the illudin precursor of irofulven by *Omphalotus* have not been attempted because of the lack of a genetic system. Our work suggests that examining monokaryons for enhanced illudin production could reveal isolates with substantially increased production. Such clones could further be increased using the enhanced production methods recently developed for *O. nidiformis* which have included process development, improving downstream processing, and improving production by the producer strain [35,36,37]. Overall, investigating haploids as an approach to finding strains with improved biosynthesis of metabolites produced by basidiomycetes is supported by this study.

While *Omphalotus* has not been genetically manipulated, genomic analysis of *Omphalotus* spp. has provided the basis of terpene production [2,72,73]. Wawrzyn et al. [72] were successful in first identifying the genes from *Omphalotus olearius* and then expressing them in *Escherichia coli*. They were able to produce twelve different products including protoilludine. Kim et al. [2] further examined *O. guepiniiformis* and identified homologous genes and performed a synteny analysis, which is likely to further understanding of the genes involved in terpene biosynthesis, including illudins. The genes Omp4, Omp6, and Omp7, for example, are components of the pathway that produces protoilludene, the precursor to illudin S, and were further studied by Yang et al. [73]. As heterologous gene expression in tumor-targeted *Salmonella* and *E. coli* strains has been used to enhance antitumor activity [74], the expression of therapeutic illudins by these strains offers a potential therapeutic delivery system, as illustrated by the heterologous expression of cytosine deaminase in *Salmonella*, which successfully converts 5-fluorocytosine to the anticancer agent 5-fluoruricil [75]. Our work shows that the cytotoxic product of *Omphalotus olivascens* that is consistent with illudin M is variably produced by monokaryons, which could offer the opportunity to genetically dissect its production by comparative genomic analysis in future studies.

## Figures and Tables

**Table 1:** Summary of the observations and measurements of the fungal strains made in this study.

Isolate	Growth MEA	Clamp Connections	Mating Type	Pigment in BCY Agar	Pigment in M9 lq (nm)	Biolum. Median Intensity	Caffeic Fold-Increase	Fluor. (nm)	Cas. Growth	Cas. Fluor.	Ab Staph	Ab Myco	468 IC50 (μL)
CC1	++++++	X		light brown	dark brown (285; 371)	8461	2.20	473	+	+	0	0	0.28
1	++	-	A1B1	light brown	light yellow (283)	512	18.80	477	-	+	0	0	0.12
2	++	-	A1B1	light brown	light yellow	4070	6.85	473	-	+	0	2 mm	0.11
3	+++++	-	AxBy	light brown	clear	7739	4.90				0	21 mm	0.023
4	++++	-	A2B2	indistinct	dark brown (285; 299; 377)	49614	3.68	471	+	o	0	15 mm	0.032
5	+++++	-	A2B2	indistinct	clear	31922	2.05						
6	++++	X		indistinct	light yellow	3980	4.41						
7a	+	-	A2B2	light brown	clear	1942	5.73	479		o			
8	+++	-	AxBy	indistinct	very light yellow	19780	2.82	467	-	+			
9	++++	-	AyBx	indistinct	very dark	15039	2.75						
10	+++++	-	AyBx	indistinct	brown	30802	3.89	471	+	+			
11	++++	-	A2B2	indistinct	brown (285; 373)	2232	18.92	471	+	+			
12	+	-	A2B2	indistinct	clear	311	19.71						
13	++++	-		indistinct	very light yellow	13822	2.14						
14	+++++	X		indistinct	very light yellow	2988	2.61						
15	+++++	X		indistinct	yellow	35792	1.83						
16	++++	-		yellowish	yellow (285; 377)	13635	1.91						
17	++++	-		indistinct	yellow	7759	5.69						
18	+	-		amber brown	very light yellow	815	4.48						
19	++++	-		indistinct	very dark brown	4896	3.72						
20	+++++	X		indistinct	brownish yellow	7072	3.54						
21	++++	-		indistinct	clear	41180	1.85						
22	++++	-		indistinct	light yellow	37826	4.02						
23	+++	-		light brown	yellow brown (283)	2582	17.22						
24	++++	-		indistinct	yellow brown	7475	15.12						
25	++++	-		indistinct	yellow brown (289)	720	22.95						
26	+	-		dark brown	very light	511	4.44						
27	++	-		dark brown	light yellow	652	18.22						
28	+++++	X		indistinct	yellow brown	7826	8.37						
29	++++	-		indistinct	yellow brown	12340	1.75						
30	++	-		brown	clear	680	8.84						
31	++	-		brown	very light	2554	8.07						
32	++	-		light brown	clear	4679	3.53						
33	+++++	X		yellowish	dark brown	26075	2.41						
34	+++	-		light brown	very light yellow	1303	10.57						
35	++	-		indistinct	yellow brown	430	4.36						
36	++	-		light brown	light yellow	230	9.43						
37	++	-		amber brown	yellow brown	347	15.03						
38	+	-		indistinct	light yellow	132	4.64						
39	++	X		amber brown	very light yellow	4964	7.07						
40	++++	-		indistinct	dark brown	28497	1.63						
41	+	-		light brown	very light	78	27.89						
42	++	-		light brown	dark brown	594	11.54						
43	+++	-		indistinct	light yellow	7759	4.14						
44	+	-		dark brown	very light	1339	5.44						
45	+	-		light brown	very lite	1102	9.56						
46	+++	-		yellowish	very lite	1310	4.81						
47	+++	-		indistinct	yellow brown	5983	2.56						
7441	+++++			light brown	dark yellow (283; 396)			465					
2305	++++			yellowish	very light yellow (285; 377)			457–461					

## Data Availability

The data are available from the researchers.

## Supplementary Materials

The following supporting information can be downloaded at https://www.mdpi.com/article/10.3390/microorganisms13010059/s1, Figure S1: Visualization of fluorescence components separated by agarose gel electrophoresis; Figure S2, Effect of heat and organic solvent extraction on fluorescence.
