# Supplementary figures and images for: Spore-Derived Isolates from a Single Basidiocarp of Bioluminescent Omphalotus olivascens Reveal Multifaceted Phenotypic and Physiological Variations
Source: Microorganisms. 2025 Jan 1;13(1):59. doi: 10.3390/microorganisms13010059 (PMC11767467; doi:10.3390/microorganisms13010059)

## Slide 1
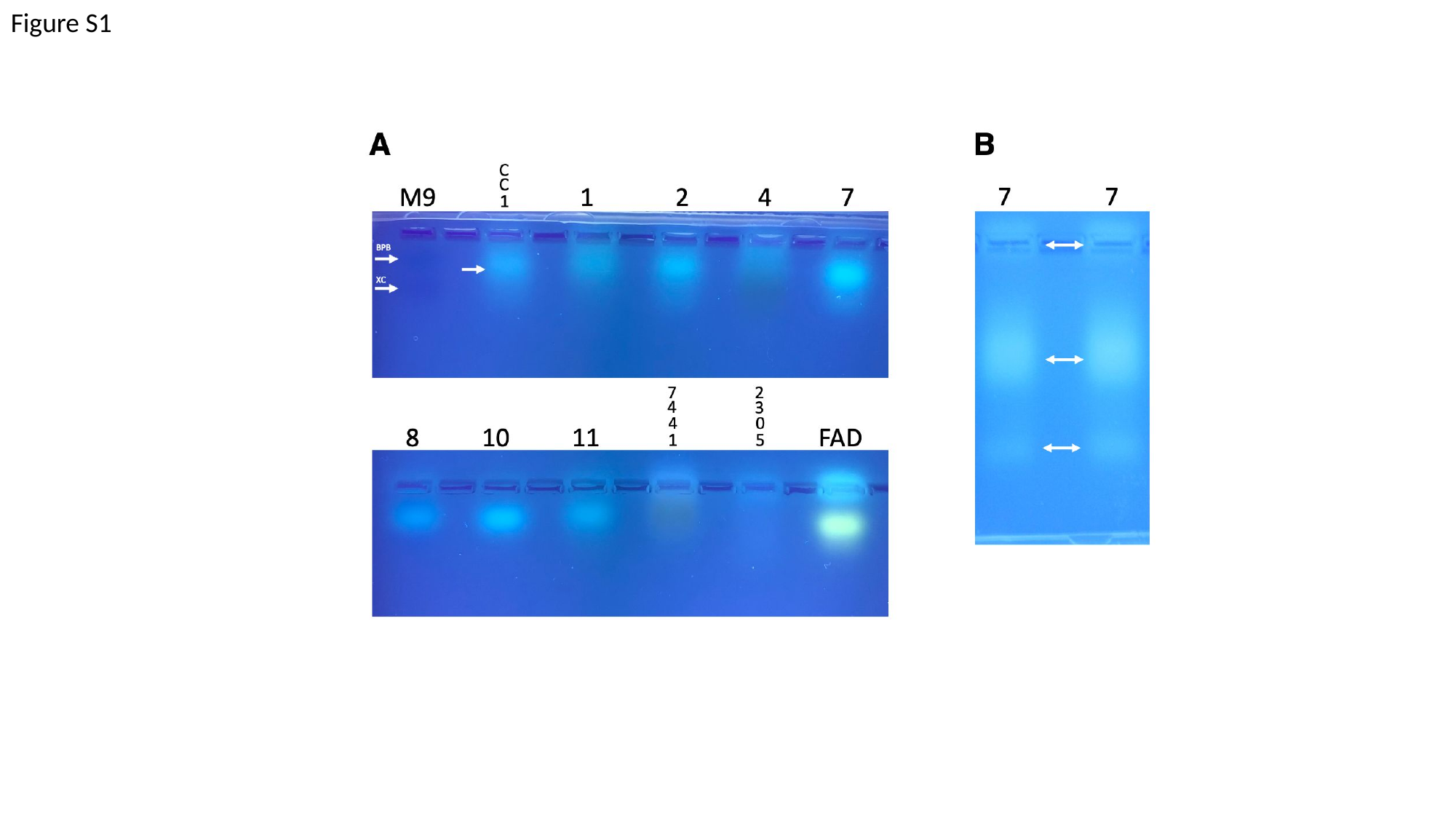

Figure S1

## Slide 2
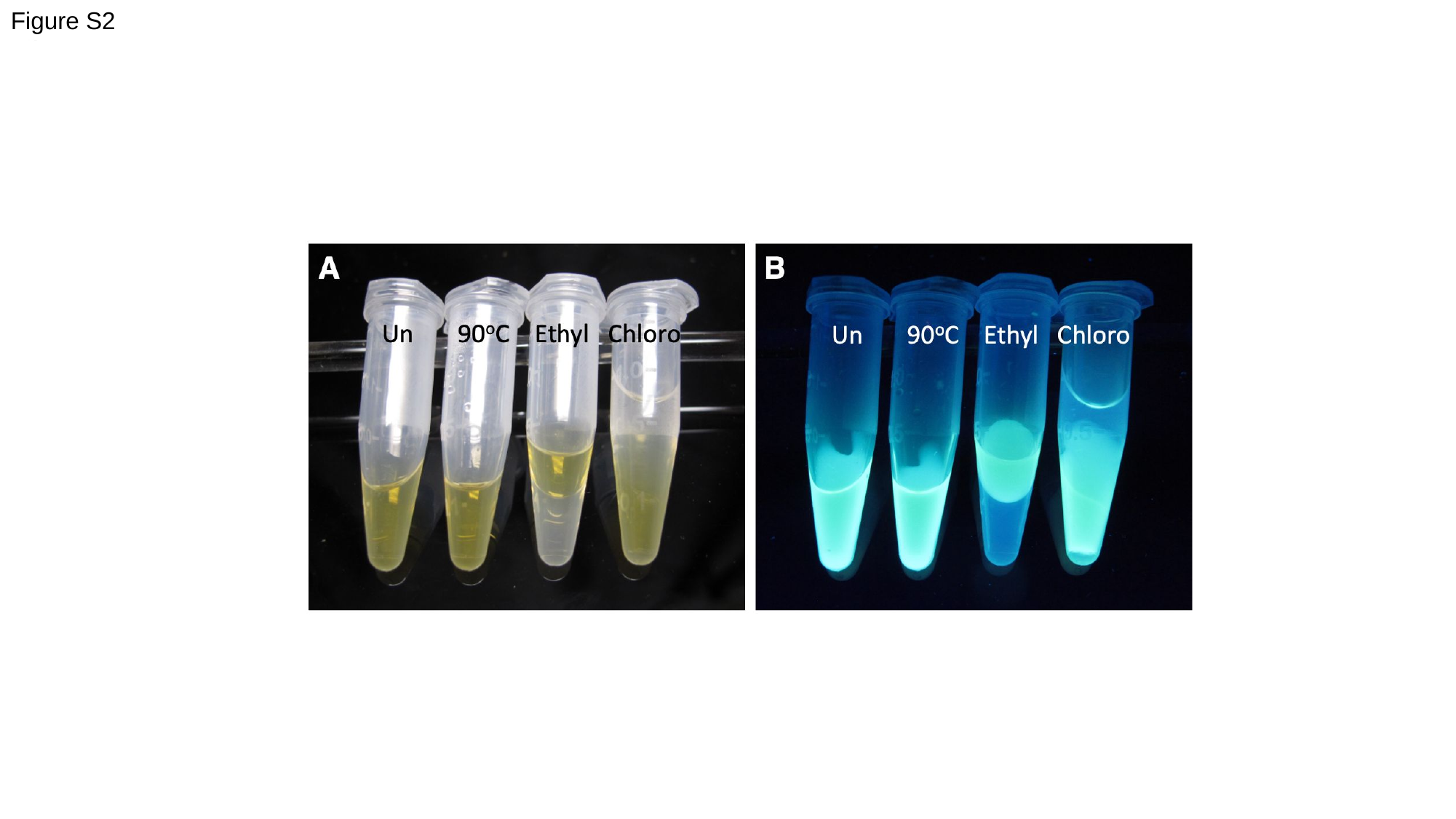

Figure S2

Supplement: Supplementary file 1 [file microorganisms-13-00059-s001.zip › Supplementary Figures.12.31.24.pptx]
